# Association Between Sleep Duration and Depressive Symptoms Among U.S. Adults: A Cross-Sectional Analysis of National Health and Nutrition Examination Survey 2021-2023

**DOI:** 10.7759/cureus.114486

**Published:** 2026-08-13

**Authors:** Sharon E Owusu, Osman A Iddrisu, Gibson Anugwom, Henry Onyeaka

**Affiliations:** 1 Biostatistics and Epidemiology, East Tennessee State University, Johnson City, USA; 2 Biostatistics and Epidemiology, Kwame Nkrumah University of Science and Technology, Kumasi, GHA; 3 Psychiatry, Baylor College of Medicine, Houston, USA; 4 Psychiatry, Harvard T.H. Chan School of Public Health, Boston, USA

**Keywords:** cross-sectional study, depression, mental health, nhanes, phq-9, sleep duration

## Abstract

Introduction

Short and long sleep durations have been associated with depressive symptoms in previous cross-sectional and prospective studies, typically demonstrating a non-linear dose-response association. This study aimed to examine the relationship between sleep duration and clinically significant depressive symptoms among U.S. adults using the redesigned National Health and Nutrition Examination Survey (NHANES) August 2021-August 2023 cycle, with sleep duration categorized into four groups, after adjustment for sociodemographic and health-related factors.

Methods

Combined NHANES 2021-2023 data (Demographics, Mental Health/Depression Screener, Sleep Disorders, Body Measures, Smoking, and Alcohol Use files) were used in this cross-sectional analysis. The nine-item Patient Health Questionnaire (PHQ-9) was used to evaluate depressive symptoms, with a score of 10 or higher indicating clinically significant depressive symptoms. Sleep time was self-reported and classified as ≤five, six, seven to eight hours (reference), and ≥nine hours. Multivariable logistic regression with survey weights, according to the NHANES complex sampling design, was used to calculate adjusted odds ratios (aORs) and 95% confidence intervals (CIs), allowing for adjustment for age, sex, race/ethnicity, education, poverty-income ratio, BMI, smoking status, and alcohol use.

Results

The analytical sample consisted of 3,727 adults with complete data on all study variables, representing approximately 148.6 million U.S. adults after application of NHANES survey weights. The overall prevalence of clinically significant depressive symptoms was 13.2%, with a range of 28.2% in adults sleeping ≤five hours to 10.2% in those sleeping seven to eight hours. Adults who slept ≤five hours had more than three times the odds of clinically significant depressive symptoms as adults who slept seven to eight hours (adjusted odds ratio or aOR 3.21, 95% CI 2.18-4.75, p<0.001); those who slept six hours had significantly elevated odds (aOR 1.59, 95% CI 1.02-2.48, p=0.041); and those who slept ≥nine hours had an elevation in odds that was not significant (aOR 1.22, 95% CI 0.96-1.54, p=0.098). There was a significant linear trend across the ordered sleep categories (OR 0.73 per category, 95% CI 0.64-0.84, p<0.001).

Conclusions

Among U.S. adults, shorter sleep duration was associated with higher odds of clinically significant depressive symptoms, with the strongest association among adults sleeping ≤five hours per night. Adults reporting ≥nine hours had higher odds, although this association was not statistically significant after adjustment. Because of the cross-sectional design of the study, causality cannot be inferred.

## Introduction

Depression is one of the most common and debilitating mental health disorders worldwide, contributing substantially to disability, reduced quality of life, and increased healthcare utilization [1]. Sleep is a fundamental physiological process that plays an important role in emotional regulation and psychological well-being. The American Academy of Sleep Medicine and the Sleep Research Society recommend that adults obtain at least seven hours of sleep per night for optimal health, as habitual short sleep has been associated with numerous adverse physical and mental health outcomes [2]. Depressive symptoms are commonly assessed using the nine-item Patient Health Questionnaire (PHQ-9), a validated screening instrument with an established cutoff for clinically significant depressive symptoms [3].

A growing body of epidemiologic evidence suggests that both insufficient and excessive sleep are associated with depression. Analyses of previous NHANES cycles consistently demonstrated a U-shaped or graded relationship, with the lowest prevalence of depressive symptoms observed among adults sleeping approximately seven to eight hours per night [4,5]. Similar findings have been reported across diverse populations, including the general U.S. population, adults with hypertension or cardiovascular disease, menopausal women, and nationally representative cohorts from other countries [6-10]. Prospective cohort studies and systematic reviews further support this association, indicating that short sleep duration is consistently associated with an increased risk of developing depression, whereas evidence for long sleep duration is less consistent [11,12]. Beyond sleep duration, sleep timing and chronotype have also been associated with depressive symptoms, suggesting that multiple dimensions of sleep contribute to mental health [13].

The relationship between sleep duration and depression has been observed internationally. Population-based studies from South Korea and China have reported patterns similar to those observed in U.S. cohorts, while longitudinal investigations indicate that shorter sleep duration predicts both incident and persistent depressive symptoms over time [14-17]. Experimental sleep restriction studies have demonstrated that inadequate sleep adversely affects emotional regulation by reducing positive affect and increasing negative mood states [18]. In addition, Mendelian randomization and genome-wide association studies provide evidence supporting a potential causal effect of short sleep duration on depression risk [19,20]. Previous investigations have also described demographic determinants of sleep duration in the U.S. population and summarized the relationship between sleep disturbances and depression among older adults [21,22].

More recently, analyses using the redesigned NHANES 2021-2023 cycle have examined related aspects of sleep and depression. Peng et al. evaluated sleep duration continuously using piecewise regression, whereas Sun et al. examined weekend catch-up sleep in relation to depressive symptoms [23,24]. The present study addresses a different analytic question by examining prespecified categories of usual weekday/workday sleep duration (≤five, six, seven to eight, and ≥nine hours) using survey-weighted multivariable logistic regression. In addition to adjusted odds ratios, we estimated model-based predicted probabilities across sleep-duration categories. This approach provides directly interpretable estimates across sleep-duration groups and facilitates comparison with earlier categorical NHANES analyses.

Therefore, we examined the association between self-reported sleep duration and clinically significant depressive symptoms among U.S. adults using NHANES 2021-2023. We hypothesized that adults reporting shorter sleep durations would have the highest odds of clinically significant depressive symptoms, with the lowest odds among those sleeping seven to eight hours per night and a smaller increase among adults reporting nine or more hours of sleep.

## Materials and methods

Study design and data source

This cross-sectional study used publicly available de-identified data from the NHANES August 2021-August 2023 cycle of the non-institutionalized civilian U.S. population [25]. The NHANES employs a complex, multistage, stratified, and clustered probability sampling design with oversampling of specific subsamples and weights, primary sampling units (PSUs), and stratum variables to generate nationally representative estimates and adjust for standard errors. Data files and documentation were retrieved from the publicly available NHANES Questionnaires, Datasets, and Related Documentation portal [25].

The following NHANES 2021-2023 component datasets were downloaded and merged using the unique respondent sequence number (SEQN): Demographic Variables and Sample Weights (DEMO_L), Mental Health/Depression Screener (DPQ_L), Sleep Disorders (SLQ_L), Body Measures (BMX_L), Smoking-Cigarette Use (SMQ_L), and Alcohol Use (ALQ_L). 

Study population

The analytical sample consisted of adults (18+ years of age) who had complete information on the outcome, exposure, and each of the covariates below. For the inclusion criteria, participants aged 18 years and older with complete data on sleep duration, depressive symptoms (PHQ-9), and all covariates included in the analysis were eligible. Individuals with missing data on any study variable were excluded, resulting in a final analytic sample of 3,727 participants. 

Outcome: depressive symptoms

Depression symptoms were assessed with the validated, widely used self-report screening instrument, the PHQ-9 (NHANES variables DPQ010-DPQ090), which is based on the nine Diagnostic and Statistical Manual of Mental Disorders (DSM-IV) criteria for major depressive disorder [3]. The scoring range for each item is 0 "not at all" to 3 "nearly every day," and the items are summed to yield a total score of 0 to 27. Using the validated cut point, participants who had a total score of 10 or more were considered to have clinically significant depressive symptoms (coded 1), and those who had a total score of less than 10 were considered not to have clinically significant depressive symptoms (coded 0) [3].

Exposure: sleep duration

The usual number of hours of sleep on weekdays or workdays (SLD012; self-reported in NHANES) was used to assess sleep duration. According to the prespecified analysis plan, sleep duration was categorized as ≤five hours, six hours, seven to eight hours, and ≥nine hours. The seven to eight hour category was selected as the reference because it falls within the range considered sufficient for most adults by the American Academy of Sleep Medicine and Sleep Research Society [2]. The remaining categories were used to distinguish very short sleep (≤five hours), shorter sleep (six hours), and long sleep (≥nine hours), while facilitating comparison with previous epidemiologic and NHANES studies [4,6,7]. The primary exposure in this analysis was usual weekday/workday sleep duration; weekend sleep duration and average weekly sleep were not included because they represent different sleep measures from the exposure examined in this analysis.

Covariates

The following covariates were included because previous studies reported associations with sleep duration and/or depressive symptoms [6,7,22]: age in years at screening (RIDAGEYR, continuous); gender (RIAGENDR: male (reference), female); race/Hispanic origin with Non-Hispanic Asian category (RIDRETH3, NHANES standard categories); education level - adults 20+ (DMDEDUC2, NHANES standard categories); ratio of family income to poverty (INDFMPIR, continuous); body mass index (BMXBMI, kg/m², continuous); smoking status (derived from smoked at least 100 cigarettes in life (SMQ020)/Do you now smoke cigarettes? (SMQ040): never, former, current); and alcohol use (derived from ever had a drink of any kind of alcohol (ALQ111)/past 12 months - how often drank alcoholic beverages (ALQ121)) [25]. Other potentially relevant factors, including marital status, employment status, medical comorbidities, diagnosed sleep disorders, and medication use, were not included in the adjusted model. 

The study is reported in accordance with the Strengthening the Reporting of Observational Studies in Epidemiology (STROBE) guidelines [26]. 

Statistical analysis

All analyses were carried out in STATA MP Version 17.0 (StataCorp LLC, College Station, TX, US), with the aid of survey (svy)-related commands to account for the NHANES complex sample design. Survey-weighted percentages and means were estimated using the NHANES Mobile Examination Center examination weights (WTMEC2YR), together with the corresponding strata and primary sampling unit (PSU) variables, in accordance with NHANES analytic guidelines. The survey weights account for the unequal probability of selection, oversampling, and nonresponse, allowing the estimates to be nationally representative of the non-institutionalized U.S. adult population. All weighted analyses were performed using the survey (svy) procedures in STATA MP Version 17.0. The NHANES weighting instructions directed that the weight from the Mobile Examination Center (WTMEC2YR) is to be used since BMI is an MEC-exam-only variable and hence was included in the adjusted model; this weight is necessary when an MEC-exam variable is used with interview-based variables [25]. The overall and sleep-duration-specific distributions of participants' characteristics were summarized descriptively, based on weighted means for continuous variables and weighted proportions for categorical variables. A survey-weighted multivariable logistic regression model was created using clinically significant depression symptoms (PHQ-9 score ≥ 10) as the outcome and sleep-duration category as the primary exposure (seven to eight hours as reference) and the covariates listed above. Odds ratios and 95% confidence intervals were calculated in the model, and a linear trend across ordered sleep categories was further tested for a dose-response gradient. Average predictive margins were calculated to estimate the average probability of clinically significant depressive symptoms for each sleep category, under the observed covariate distribution. Statistical significance was defined as a two-sided p-value <0.05. The associations mentioned herein should be viewed as cross-sectional, not causal associations, since NHANES is a cross-sectional survey.

Ethical considerations

This study was a secondary analysis of publicly available, de-identified data from the National Health and Nutrition Examination Survey (NHANES). The NHANES protocol was approved by the National Center for Health Statistics (NCHS) Research Ethics Review Board, and all participants provided written informed consent before participation. Because the present study used publicly available de-identified data, additional institutional review board (IRB) approval and informed consent were not required [25]. 

## Results

Participant characteristics

The NHANES 2021-2023 merged dataset consisted of 11,933 participants, of which 8,153 were adults (age 18 or older). A final analytic sample of 3,727 adults (45.7% of the 8,153 eligible adults) was included in the analysis. After application of survey weights, this sample represented approximately 148.6 million U.S. adults. The analytic sample was created by excluding participants with missing PHQ-9 data (n=2,698) and, among the remaining participants, those with missing data on sleep-duration category, education, poverty-income ratio, body mass index, smoking status, or alcohol use (n=1,728). The highest proportion of exclusions was due to missing PHQ-9 data, accounting for 61.0% of all excluded participants. 

The weighted participant characteristics are summarized in Table 1.

**Table 1:** Descriptive characteristics of U.S. adults by sleep duration category, NHANES 2021–2023 (unweighted N=3,727) NHANES, National Health and Nutrition Examination Survey; SE, standard error. Frequencies are presented as unweighted counts (n), and percentages are survey-weighted estimates accounting for the NHANES complex sampling design. Continuous variables are presented as weighted means (SE). The overall weighted prevalence of depressive symptoms (PHQ-9 score ≥10) was 13.2%.

Characteristic	≤5 h (n = 299)	6 h (n = 308)	7–8 h (n = 2163)	≥9 h (n = 957)
Age in years, mean (SE)	51.17 (1.12)	47.96 (0.76)	48.81 (0.70)	49.19 (0.90)
Sex, n (weighted %)				
Male	140 (52.8%)	147 (46.6%)	981 (49.4%)	402 (47.8%)
Female	159 (47.2%)	161 (53.4%)	1182 (50.6%)	555 (52.2%)
Race/ethnicity, n (weighted %)				
Mexican American	19 (8.5%)	22 (6.9%)	115 (5.8%)	73 (7.3%)
Other Hispanic	32 (10.6%)	35 (12.1%)	185 (7.3%)	92 (9.1%)
Non-Hispanic White	137 (44.1%)	162 (55.2%)	1432 (66.3%)	581 (63.3%)
Non-Hispanic Black	62 (19.6%)	53 (16.0%)	182 (7.5%)	119 (11.3%)
Non-Hispanic Asian	16 (6.6%)	18 (5.5%)	110 (6.2%)	21 (2.8%)
Other/Multiracial	33 (10.6%)	18 (4.4%)	139 (6.8%)	71 (6.3%)
Education, n (weighted %)				
Less than 9th grade	8 (2.1%)	12 (2.4%)	52 (1.8%)	48 (4.2%)
9–11th grade	36 (9.9%)	23 (6.4%)	119 (4.4%)	91 (8.2%)
High school graduate/GED	77 (32.1%)	70 (30.8%)	364 (20.4%)	223 (29.3%)
Some college/AA degree	110 (33.7%)	98 (33.6%)	643 (29.6%)	321 (33.7%)
College graduate or above	68 (22.2%)	105 (26.8%)	985 (43.8%)	274 (24.6%)
Poverty-income ratio, mean (SE)	2.73 (0.12)	2.78 (0.15)	3.34 (0.11)	2.68 (0.12)
BMI, kg/m², mean (SE)	31.52 (0.46)	30.14 (0.59)	29.48 (0.38)	29.93 (0.29)
Smoking status, n (weighted %)				
Never	144 (53.4%)	174 (56.1%)	1321 (64.1%)	513 (57.6%)
Former	71 (22.3%)	77 (21.1%)	579 (23.4%)	282 (26.5%)
Current	84 (24.3%)	57 (22.8%)	263 (12.5%)	162 (15.9%)
Alcohol use, n (weighted %)				
Never drinker	27 (9.0%)	32 (7.4%)	138 (6.7%)	116 (11.5%)
Former/none past 12 months	61 (19.4%)	53 (10.9%)	289 (10.9%)	175 (16.1%)
Current drinker	211 (71.6%)	223 (81.7%)	1736 (82.4%)	666 (72.4%)
Clinically significant depressive symptoms (PHQ-9 ≥10), n (weighted %)	86 (28.2%)	59 (17.6%)	215 (10.2%)	139 (14.4%)

The mean age of adults who reported ≤five hours of sleep was 51.2 years, higher than the mean ages of the other three groups, and the current smoking prevalence was highest at 24.3% among the four groups. The mean BMI (kg/m²) of adults who reported ≤five hours of sleep was the highest (31.5 kg/m²) among the four groups. The percentage of college-educated adults (43.8%) and the mean poverty-income ratio (3.34) were the highest among those who slept seven to eight hours. The weighted prevalence of clinically significant depressive symptoms was 28.2% among adults reporting ≤five hours of sleep, 17.6% among those reporting six hours, 10.2% among those reporting seven to eight hours, and 14.4% among those reporting ≥nine hours.

Association between sleep duration and depressive symptoms

The adults who slept ≤five hours had more than three times the odds of clinically significant depressive symptoms than those who slept seven to eight hours (adjusted odds ratio or aOR 3.21, 95% CI 2.18-4.75, p<0.001) in the fully adjusted survey-weighted logistic regression model. Adults sleeping six hours also had significantly elevated odds (aOR 1.59, 95% CI 1.02-2.48, p=0.041). There were modestly elevated but not significant odds ratio (aOR 1.22, 95% CI 0.96-1.54, p=0.098) for adults sleeping ≥nine hours. Older age (aOR 0.98 per year, 95% CI 0.97-0.98, p<0.001) and higher poverty-income ratio (aOR 0.77 per unit, 95% CI 0.71-0.84, p<0.001) were independently associated with lower odds of depressive symptoms, while female sex (aOR 1.44, 95% CI 1.09-1.90, p=0.014) and higher BMI (aOR 1.02 per kg/m², 95% CI 1.01-1.04, p=0.013) were independently associated with higher odds as seen in Table 2.

**Table 2:** Adjusted odds ratios for clinically significant depressive symptoms (PHQ-9 ≥ 10) by sleep-duration category, NHANES 2021-2023 NHANES, National Health and Nutrition Examination Survey; OR, odds ratio; CI, confidence interval; PHQ-9, Patient Health Questionnaire-9. Odds ratios were estimated using a survey-weighted multivariable logistic regression model including sleep duration, age, sex, race/ethnicity, education, poverty-income ratio, body mass index, smoking status, and alcohol use. The t statistic and corresponding p-value are from the survey-weighted logistic regression model. Design df = 15 (30 primary sampling units, 15 strata).  Analytical sample: n=3,727 participants.

Variable	Adjusted OR (95% CI)	t	p-value
Sleep duration			
≤5 hours	3.21 (2.18–4.75)	6.38	<0.001
6 hours	1.59 (1.02–2.48)	2.24	0.041
7–8 hours	Reference	—	—
≥9 hours	1.22 (0.96–1.54)	1.77	0.098
Age (years)	0.98 (0.97–0.98)	-7.34	<0.001
Sex			
Male	Reference	—	—
Female	1.44 (1.09–1.90)	2.78	0.014
Race/Ethnicity			
Non-Hispanic White	Reference	—	—
Mexican American	0.72 (0.38–1.36)	-1.11	0.283
Other Hispanic	0.72 (0.51–1.03)	-1.95	0.070
Non-Hispanic Black	0.70 (0.44–1.11)	-1.66	0.119
Non-Hispanic Asian	0.42 (0.18–1.01)	-2.10	0.053
Other/Multiracial	1.03 (0.75–1.42)	0.23	0.822
Education			
High school graduate/GED	Reference	—	—
Less than 9th grade	1.29 (0.57–2.91)	0.66	0.519
9–11th grade	0.99 (0.66–1.48)	-0.06	0.952
Some college/AA degree	0.99 (0.71–1.39)	-0.06	0.954
College graduate or above	0.99 (0.61–1.61)	-0.04	0.970
Poverty-income ratio	0.77 (0.71–0.84)	-6.55	<0.001
Body mass index (kg/m²)	1.02 (1.01–1.04)	2.80	0.013
Smoking status			
Never	Reference	—	—
Former	0.91 (0.61–1.35)	-0.51	0.617
Current	1.46 (0.94–2.29)	1.83	0.088
Alcohol use			
Never drinker	Reference	—	—
Former/none past 12 months	1.32 (0.78–2.23)	1.13	0.275
Current drinker	1.00 (0.58–1.73)	0.00	1.000

There was a significant linear trend across the four ordered sleep groups (OR 0.73 per category, 95% CI 0.64-0.84, p<0.001), suggesting a dose-response effect rather than just an effect of the shortest sleep group.

**Figure 1:**
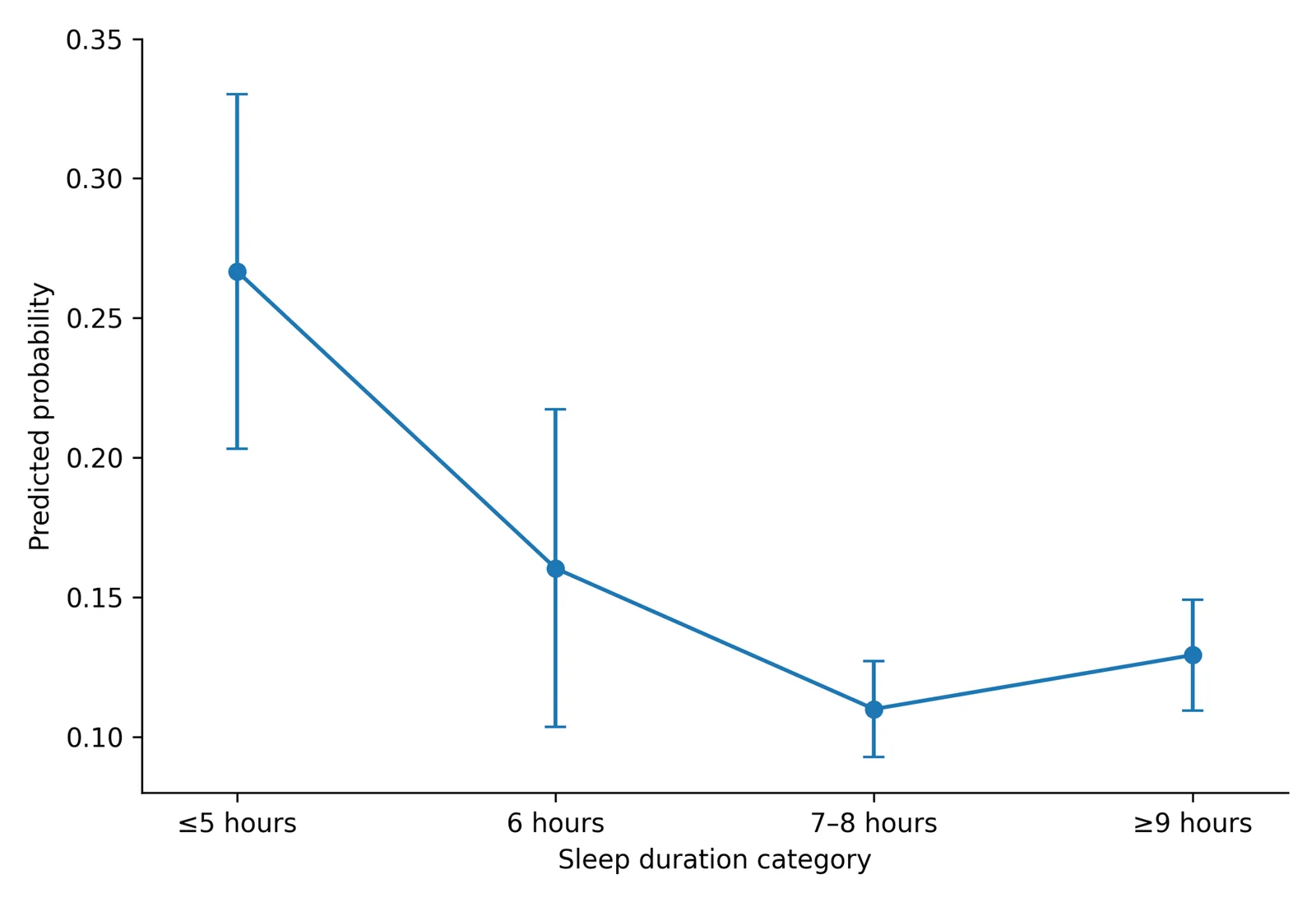
Model-based average predictive probability of clinically significant depressive symptoms (PHQ-9≥10) by sleep-duration category, with 95% confidence intervals, NHANES 2021-2023. NHANES, National Health and Nutrition Examination Survey; PHQ, Patient Health Questionnaire.

The average predictive margins from this same model showed a corresponding gradient in the model-implied probability of clinically significant depressive symptoms: 26.7% (95% CI 20.3-33.0%) for ≤five hours, 16.0% (95% CI 10.3-21.7%) for six hours, 11.0% (95% CI 9.3-12.7%) for seven to eight hours, and 12.9% (95% CI 11.0-14.9%) for ≥nine hours (Figure 1), visually capturing the pattern of the odds ratios and suggesting a slight upturn at the end of the long-sleep distribution.

## Discussion

In this nationally representative study of U.S. adults using the redesigned NHANES 2021-2023 cycle, short sleep duration was independently associated with clinically significant depressive symptoms in a dose-response pattern. Adults reporting ≤five hours of sleep had more than three times the odds of clinically significant depressive symptoms compared with those sleeping seven to eight hours, while those reporting six hours of sleep also had significantly higher odds. The association for adults sleeping ≥nine hours was in the same direction but was not statistically significant. Most of the association in our sample came from the short-sleep end of the distribution, yielding a partial U-shaped pattern.

Our findings are consistent with previous NHANES analyses showing that adults with shorter sleep duration are more likely to report depressive symptoms [6,7]. The association for very short sleep in our study (aOR 3.21) was stronger than that reported in earlier NHANES studies and larger than the pooled estimate from prospective cohort studies [4]. This may reflect differences in how sleep duration was categorized. We used a stricter definition of very short sleep (≤five hours) and a narrower reference group (seven to eight hours), which may have better distinguished adults with substantial sleep deprivation from those with recommended sleep durations. Changes in sleep habits and mental health following the COVID-19 pandemic may also have contributed to the stronger association observed in the 2021-2023 cycle.

Long sleep duration showed a different pattern. Although adults sleeping ≥nine hours had higher odds of depressive symptoms, the association was no longer statistically significant after adjustment. Previous NHANES studies have reported significant associations for long sleep [6,7], but systematic reviews have found this relationship to be less consistent than that observed for short sleep [11]. The wider confidence interval in our study may reflect the smaller number of participants in the ≥nine-hour category or the greater clinical heterogeneity among adults who report prolonged sleep [27].

Our results also fit well with recent analyses of the redesigned NHANES cycle. Peng et al. reported a nonlinear relationship between sleep duration and depression when sleep was analyzed as a continuous variable [23]. Sun et al. showed that moderate weekend catch-up sleep was associated with the lowest risk of depressive symptoms, suggesting that sleep regularity may be important in addition to sleep duration [24]. Although these studies addressed different aspects of sleep, the overall pattern is similar. Together, they suggest that multiple dimensions of sleep should be considered when evaluating depression risk.

The stronger association observed among short sleepers may be understood in the context of evidence linking insufficient sleep with impaired emotional regulation. Experimental studies have shown that sleep restriction reduces positive affect, increases negative mood, and impairs emotional regulation [18], which may help explain the higher odds observed among adults reporting ≤five and six hours of sleep. Longitudinal studies indicate that the relationship is likely bidirectional, with inadequate sleep increasing the risk of future depressive symptoms while depression itself can disrupt normal sleep [22,28]. Mendelian randomization studies provide additional evidence that short sleep may play a causal role in depression [20]. These findings may help explain why the association in our study was strongest among short sleepers, whereas the weaker and non-significant association among long sleepers may reflect different or more heterogeneous underlying pathways. Sleep duration is only one component of sleep health, and factors such as chronotype and sleep timing have also been independently linked to depression [13,29].

Strengths

This study has several strengths. NHANES uses a large, nationally representative sample and standardized data collection procedures. The analyses accounted for the complex survey design using survey weights, improving the generalizability of the findings to the U.S. adult population. Depressive symptoms were assessed using the PHQ-9, a well-validated screening instrument with an established cutoff for clinically significant depressive symptoms [3]. In addition, this study used the redesigned NHANES 2021-2023 cycle with prespecified sleep-duration categories and adjustment for several sociodemographic, behavioral, and health-related factors.

Limitations

Due to the cross-sectional design of this study, causal or temporal relationships cannot be established. Sleep duration was self-reported and assessed only on weekdays/workdays rather than objectively measured; therefore, it may not fully reflect total weekly sleep patterns, weekend sleep, or compensatory weekend catch-up sleep. The PHQ-9 is a screening instrument rather than a clinical diagnosis of major depressive disorder. This study did not include measures of sleep timing, chronotype, or sleep midpoint. Residual confounding remains possible because other potentially relevant factors, including marital and employment status, medical comorbidities, diagnosed sleep disorders, and medication use, were not included in the adjusted model. Only 45.7% of eligible adults had complete data for all study variables. Most exclusions resulted from missing PHQ-9 data (61.0% of all exclusions). Although complete-case analysis was used, selection bias cannot be excluded if the missing data were not completely at random.

## Conclusions

In this nationally representative sample of U.S. adults, shorter sleep duration was independently associated with higher odds of clinically significant depressive symptoms, with the strongest association observed among adults sleeping ≤five hours per night. Although adults reporting ≥nine hours of sleep had higher odds of depressive symptoms, the association was not statistically significant after adjustment. Because of the cross-sectional design, causality cannot be inferred. Future longitudinal studies are needed to better understand the temporal relationship between sleep duration and depressive symptoms.
